# Supplementary material for: Early life inflammation is associated with spinal cord excitability and nociceptive sensitivity in human infants
Source: Nat Commun. 2022 Jul 8;13:3943. doi: 10.1038/s41467-022-31505-y (PMC9270448; doi:10.1038/s41467-022-31505-y)
Supplement: Supplementary file 1 — Supplementary Information [file 41467_2022_31505_MOESM1_ESM.pdf]

Early life inflammation is associated with spinal cord excitability and nociceptive sensitivity in human infants

Maria M Cobo, Gabrielle Green, Foteini Andritsou, Luke Baxter, Ria Evans Fry, Annika Grabbe, Deniz Gursul, Amy Hoskin, Gabriela Schmidt Mellado, Marianne van der Vaart, Eleri Adams, Aomesh Bhatt, Franziska Denk, Caroline Hartley, Rebeccah Slater

Supplementary information

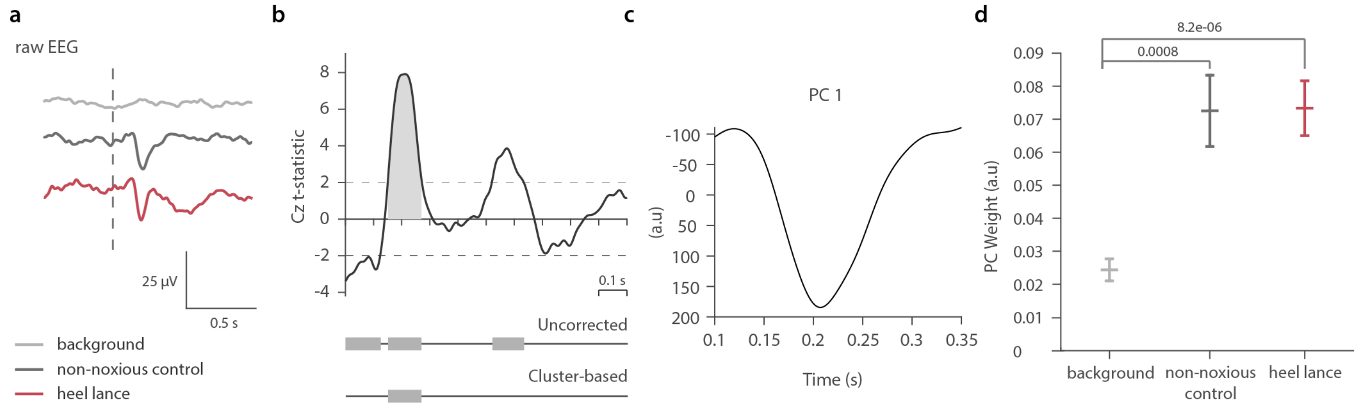

Supplementary Figure 1. Characterisation of the tactile-evoked early potential during a heel lance and a tactile stimulus. **a** Average raw EEG traces during background activity and in response to a tactile stimulus (non-noxious control) and a heel lance showing an evoked potential around 250 ms post stimulus (n=54 neonates). Dashed line indicates the point of stimulation. **b** t-statistics from the comparison of the noxious-evoked brain activity during noxious and tactile stimulation (combined) and during background brain activity (n=54). Dashed lines indicate the t-statistic threshold for cluster significance, set as the 97.5 percentile of the permuted data. The grey bars indicate time periods outside of the t-statistic threshold and the significant time window identified with the cluster analysis is illustrated by the grey shading area. **c** Principal component analysis was conducted in the time window 100-350 ms after the stimuli and the characterised waveform with a positive peak was similar to the waveform previously described in the literature [1–4]. **d** First principal component weights were significantly higher following tactile stimulus and heel lance compared with background brain activity (n=54). *P*-values were calculated using a repeated measures ANOVA with multiple comparisons and adjusted by the Bonferroni method. Error bars indicate mean ± standard error. a.u: arbitrary units. Source data are provided as a Source Data file.

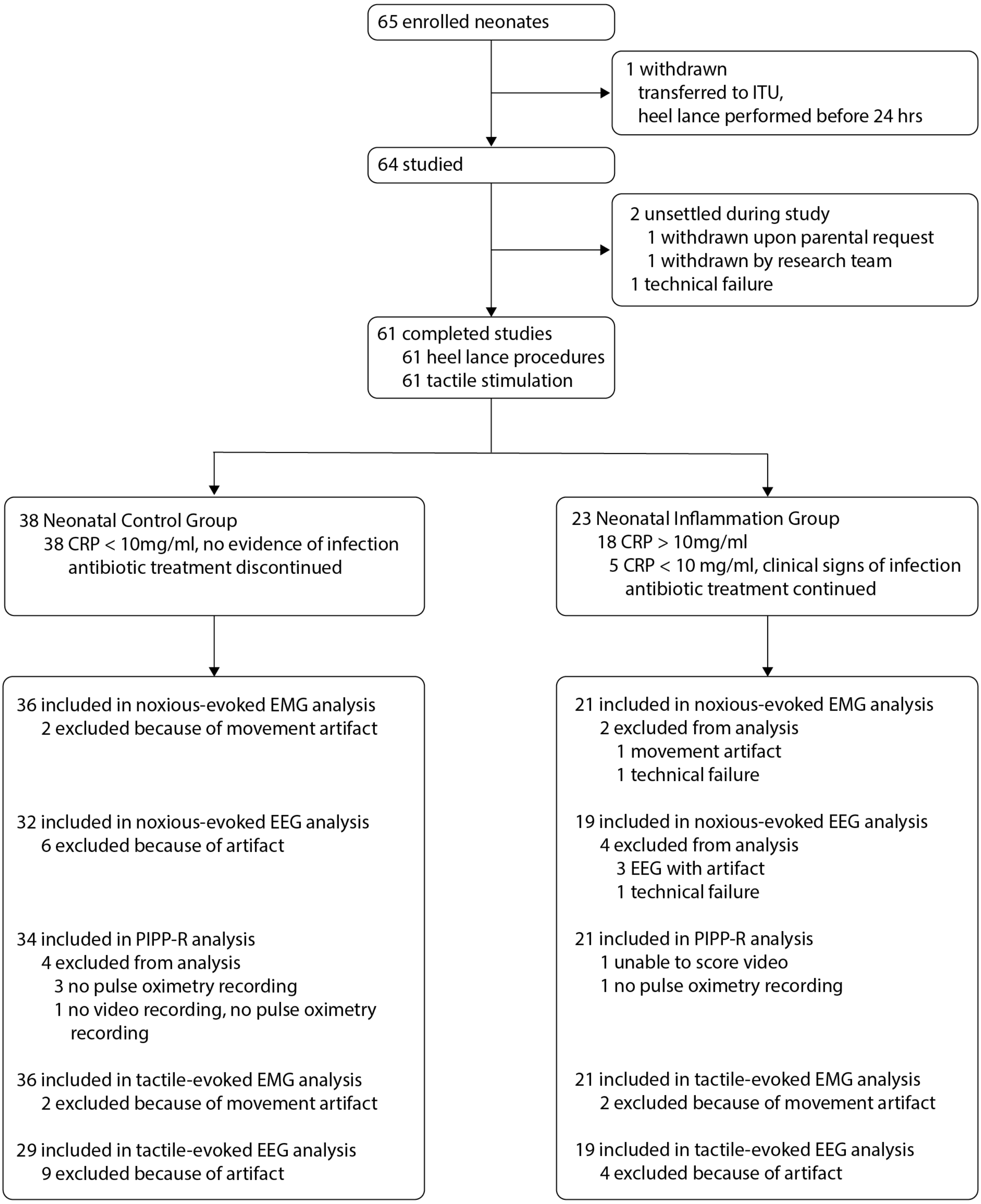

Supplementary Figure 2. Hypothesis-testing study profile. A total of 65 neonates were enrolled in the hypothesis-testing study. The diagram shows the details of the datasets included in the final analysis for each outcome.

Supplementary Table 1. Relationship between CRP levels (dichotomised and continuous) and noxious-evoked responses.

	EMG	EEG	Brow bulge	Eye squeeze	Nasolabial furrow	Heart rate change	Oxygen saturation change
**T-test**	1.74 (0.048)	2.4 (0.011)	0.53 (0.29)	0.53 (0.29)	0.72 (0.23)	-0.74 (0.77)	0.62 (0.30)
**Correlation**	0.28 (0.028)	0.31 (0.023)	0.05 (0.34)	0.03 (0.39)	0.07 (0.31)	-0.11 (0.79)	0.14 (0.13)

Brow bulge, eye squeeze, nasolabial furrow, heart rate change and oxygen saturation change correspond to the individual components of the PIPP-R scores (hypothesis-testing study).
T-test: t-statistics and one-sided uncorrected p-values (parentheses) are presented for each metric. Correlation: Pearson correlation coefficients and one-sided uncorrected p-values (parentheses) are presented for each metric.

References

1. Fabrizi, L. *et al.* A shift in sensory processing that enables the developing human brain to discriminate touch from pain. *Curr. Biol.* **21**, 1552–1558 (2011).
2. Slater, R. *et al.* Evoked potentials generated by noxious stimulation in the human infant brain. *Eur. J. pain* **14**, 321–326 (2010).
3. Hartley, C. *et al.* Nociceptive brain activity as a measure of analgesic efficacy in infants. *Sci. Transl. Med.* **9**, (2017).
4. Verriotis, M. *et al.* Mapping cortical responses to somatosensory stimuli in human infants with simultaneous near-infrared spectroscopy and event-related potential recording. *eNeuro* **3**, 663–673 (2016).
