# Supplementary material for: Early life inflammation is associated with spinal cord excitability and nociceptive sensitivity in human infants
Source: Nat Commun. 2022 Jul 8;13:3943. doi: 10.1038/s41467-022-31505-y (PMC9270448; doi:10.1038/s41467-022-31505-y)
Supplement: Supplementary file 3 — Reporting Summary [file 41467_2022_31505_MOESM3_ESM.pdf]

nature portfolio

Corresponding author(s): Rebeccah Slater

Last updated by author(s): May 17, 2022

Reporting Summary

Nature Portfolio wishes to improve the reproducibility of the work that we publish. This form provides structure for consistency and transparency in reporting. For further information on Nature Portfolio policies, see our Editorial Policies and the Editorial Policy Checklist.

Statistics

For all statistical analyses, confirm that the following items are present in the figure legend, table legend, main text, or Methods section.

n/a	Confirmed	
☐	☒	The exact sample size (n) for each experimental group/condition, given as a discrete number and unit of measurement
☐	☒	A statement on whether measurements were taken from distinct samples or whether the same sample was measured repeatedly
☐	☒	The statistical test(s) used AND whether they are one- or two-sided<br>*Only common tests should be described solely by name; describe more complex techniques in the Methods section.*
☐	☒	A description of all covariates tested
☐	☒	A description of any assumptions or corrections, such as tests of normality and adjustment for multiple comparisons
☐	☒	A full description of the statistical parameters including central tendency (e.g. means) or other basic estimates (e.g. regression coefficient) AND variation (e.g. standard deviation) or associated estimates of uncertainty (e.g. confidence intervals)
☐	☒	For null hypothesis testing, the test statistic (e.g. F, t, r) with confidence intervals, effect sizes, degrees of freedom and P value noted<br>*Give P values as exact values whenever suitable.*
☒	☐	For Bayesian analysis, information on the choice of priors and Markov chain Monte Carlo settings
☒	☐	For hierarchical and complex designs, identification of the appropriate level for tests and full reporting of outcomes
☐	☒	Estimates of effect sizes (e.g. Cohen's d, Pearson's r), indicating how they were calculated

Our web collection on statistics for biologists contains articles on many of the points above.

Software and code

Policy information about availability of computer code

Data collection	Electrophysiological activity was recorded using CURRY scan 7 neuroimaging suite (Compumedics Neuroscan). Oxygen saturation and heart rate were acquired using an IntelliVue MX800 Philips patient monitor and vital signs were continuously downloaded from the monitor using ixTrend software (ixitos GmbH, Germany).
Data analysis	Power calculations were performed in G*Power v3.1. Data analysis was performed using MATLAB_R2020a (MathWorks) and R version 3.6.3 (The R Project for Statistical Computing). The magnitude of noxious-evoked brain activity in response to the clinically-required procedure was calculated using a template of noxious-evoked brain activity, previously validated for experimental and clinical stimuli (ref 37 in the main manuscript). Statistical significance was assessed non-parametrically via permutation testing with using the PALM toolbox (https://github.com/andersonwinkler/PALM). The effect size estimations were calculated using the DABEST web application (https://www.estimationstats.com).

For manuscripts utilizing custom algorithms or software that are central to the research but not yet described in published literature, software must be made available to editors and reviewers. We strongly encourage code deposition in a community repository (e.g. GitHub). See the Nature Portfolio guidelines for submitting code & software for further information.

Data

Policy information about availability of data

All manuscripts must include a data availability statement. This statement should provide the following information, where applicable:

- Accession codes, unique identifiers, or web links for publicly available datasets
- A description of any restrictions on data availability
- For clinical datasets or third party data, please ensure that the statement adheres to our policy

Source data to produce Figures 2, 3 and Supplementary Figure 1 are provided with the paper. The data that support the findings of this study are available from the corresponding author (rebeccah.slater@paediatrics.ox.ac.uk) within three months upon request. Due to ethical restrictions, we consider it appropriate to monitor the access and usage of the data as it includes highly sensitive information.

Field-specific reporting

Please select the one below that is the best fit for your research. If you are not sure, read the appropriate sections before making your selection.

[x] Life sciences [ ] Behavioural & social sciences [ ] Ecological, evolutionary & environmental sciences

For a reference copy of the document with all sections, see nature.com/documents/nr-reporting-summary-flat.pdf

Life sciences study design

All studies must disclose on these points even when the disclosure is negative.

Sample size

The first study is a hypothesis-testing study. The principal hypothesis was that neonatal inflammation causes increased spinal cord excitability and hyperalgesia in response to noxious stimulation and a power calculation was performed to determine the sample size required to test this principal hypothesis.

The mean (SD) brain activity evoked by a heel lance in a cohort of healthy term infants is 0.72 (0.69). A sample size of 56 neonates (2:1 Neonatal Control Group to Neonatal Inflammation Group allocation ratio) would be required to observe a 70% increase in noxious-evoked brain activity with a two-sample t-test (80% power and a one-sided 5% significance level). A 70% increase in the outcome measures (noxious-evoked reflex withdrawal and brain activity) in the Neonatal Inflammation Group was considered to be clinically significant as this effect has been reported for inflammation induced hyperalgesia in adults. We allowed for a total sample size of 65 neonates to account for 15% of loss due to technical failures or clinical ineligibility (it was expected that after enrollment some infants would require recannulation for antibiotic administration therefore precluding the need to perform a heel lance). We also calculated the sample size that would be required to achieve adequate power to observe a significant difference in reflex withdrawal activity between the two groups. The mean (SD) RMS of the reflex withdrawal in a cohort of healthy term infants is 23.3 (17.7). Assigning the same assumptions as for the noxious-evoked brain activity a lower sample size of 36 neonates would be required for this measure. Therefore, a sample size of 65 neonates ensured that adequate power could be achieved for both outcome measures.

A power calculation was not performed for the second hypothesis because it had the purpose of supporting and providing greater insight to the primary results. The same sample of 65 neonates recruited for the main hypothesis testing study was used for the second hypothesis to maximize the use of the collected data. The observed results can be used to calculate the power for a future prospective study.

Sample size calculations were not performed for the exploratory study as no hypotheses were being tested.

Data exclusions

In the hypotheses-testing study, 2 out of 38 noxious and tactile-evoked EMG responses were rejected for gross movement artefacts from the Neonatal Control Group and 2 out of 23 traces (1 with artifact and 1 due to technical failure) were rejected from the Neonatal Inflammation Group. A total of 57 participants were included in the final noxious and tactile evoked EMG data analysis.

EEG traces were rejected for gross movement artefacts; 6 out of 38 noxious-evoked EEG traces were rejected from the Neonatal Control Group and 4 out of 23 noxious-evoked traces (3 with artifact and 1 due to technical failure) were rejected from the Neonatal Inflammation Group. Additionally, 9 out of 38 tactile-evoked EEG epochs with artifact were rejected from the Neonatal Control Group and 4 out of 23 tactile-evoked traces with artifacts were rejected from the Neonatal Inflammation Group for the EEG analysis.

All data was included for the exploratory study EEG analysis.

Replication

In the hypotheses-testing study, the noxious-evoked brain activity measures that were recorded in the Neonatal Control Group in response to the heel lance, had a range of values similar to published previous work from our group (range: 0.09 – 0.77). Likewise, the magnitudes of the muscle reflex withdrawal evoked by the heel lances in the Neonatal Control Group were similar to those evoked by heel lance in previous publications (range: 1 - 80 µV). The effect of early onset inflammation on pain related responses was not replicated because recruitment and clinical research were suspended during the COVID-19 pandemic response. As noted in the manuscript the observations from the exploratory study warrant further investigation with a prospective study.

Randomization

Allocation of participants into experimental groups was not random. In the hypotheses-testing study participants were grouped into the Neonatal Inflammation Group based on the blood test results (24 hours after the first dose of antibiotics): CRP > 10 mg/l, or other laboratory or clinical evidence of infection. Participant were grouped into the Neonatal Control group when CRP < 10 mg/l and clinically asymptomatic. The threshold of 10 mg/l was selected because in our institution, prophylactic antibiotic treatment was discontinued 36-hours after the first

	dose if the CRP was below 10 mg/l and there were no other clinical or laboratory signs of infection.<br><br>In the exploratory study, neonates receiving 5 days of antibiotics and due a blood test tot check CRP levels 84-96 hours after the first dose of antibiotics were assigned to the Neonatal Antibiotic Treatment group. Age-matched neonates with no previous evidence of infection or antibiotic treatment who required a blood test for routine newborn screening were included in the Neonatal Antibiotic Control Group.
Blinding	Researchers were blinded to the participants' clinical status at the time of the study and neonates were assigned to the Neonatal Control Group or Neonatal Inflammation Group after the study was complete based on clinical assessment, CRP levels within 24 hours from presentation of risk factors, other laboratory results and the clinical decision to continue with intravenous antibiotics to complete a minimum of a 5-day course.

Reporting for specific materials, systems and methods

We require information from authors about some types of materials, experimental systems and methods used in many studies. Here, indicate whether each material, system or method listed is relevant to your study. If you are not sure if a list item applies to your research, read the appropriate section before selecting a response.

Materials & experimental systems

n/a	Involved in the study
☒	☐ Antibodies
☒	☐ Eukaryotic cell lines
☒	☐ Palaeontology and archaeology
☒	☐ Animals and other organisms
☐	☒ Human research participants
☒	☐ Clinical data
☒	☐ Dual use research of concern

Methods

n/a	Involved in the study
☒	☐ ChIP-seq
☒	☐ Flow cytometry
☒	☐ MRI-based neuroimaging

Human research participants

Policy information about studies involving human research participants

Population characteristics	Population characteristics hypotheses-testing study: gestational age (GA) at birth > 36 weeks (range: 36 - 42 weeks) . Postnatal age (PNA) range 1 to 3 days. Male to female ratio 36/25.<br>Population characteristics exploratory study: gestational age (GA) at birth > 36 weeks (range 36 - 42 weeks) . Postnatal age (PNA) range 4 to 6 days. Male to female ratio 10/10.<br>All infants were assessed as clinically stable and were not ventilated at the time of the study.<br>Infants with IUGR, IVH grade 3 or above or any other neurological abnormalities were not eligible to take part in these studies.
Recruitment	Screening was conducted in the neonatal unit and maternity wards. Infants requiring a blood test and complying with the study inclusion criteria were identified. Members of the clinical team responsible for the patient determined whether it was appropriate to approach parents about the research study. Parents were approached and asked if they wanted to participate in the research. The study was then explained and a PIL given, if interested. Parents had a minimum of one hour to consider taking part in the study. All questions were addressed by the research team members. Parents willing to take part signed a consent form. Timing of the study was agreed in coordination with the parents and the clinical teams.<br><br>Potential self-selection biases include the recruitment of participants in a single centre in the UK and from an age group limited to neonates from birth to 72 hours old. Our results cannot be generalised to other age categories or to suspected sepsis that has a late onset in this population.
Ethics oversight	Ethical approval was obtained from the National Research Ethics Service, UK (reference 12/SC/0447).

Note that full information on the approval of the study protocol must also be provided in the manuscript.
